# Resveratrol Downmodulates Neutrophil Extracellular Trap (NET) Generation by Neutrophils in Patients with Severe COVID-19

**DOI:** 10.3390/antiox11091690

**Published:** 2022-08-29

**Authors:** Milena M. de Souza Andrade, Vinicius N. C. Leal, Iara G. Fernandes, Sarah C. Gozzi-Silva, Danielle R. Beserra, Emily A. Oliveira, Franciane M. E. Teixeira, Tatiana M. Yendo, Maria da Glória T. Sousa, Walcy R. Teodoro, Luana de M. Oliveira, Ricardo W. Alberca, Valéria Aoki, Alberto J. S. Duarte, Maria N. Sato

**Affiliations:** 1 Laboratory of Dermatology and Immunodeficiencies, LIM-56, Department of Dermatology, Tropical Medicine Institute of São Paulo, University of São Paulo Medical School, São Paulo 05403-000, Brazil; 2 Department of Immunology, Institute of Biomedical Sciences, University of São Paulo, São Paulo 05508-000, Brazil; 3 Hospital das Clínicas of the University of São Paulo (HCFMUSP), University of São Paulo, São Paulo 05403-000, Brazil; 4 Laboratory of Mycology, LIM-53, Department of Dermatology, Tropical Medicine Institute of São Paulo, University of São Paulo Medical School, São Paulo 05403-000, Brazil; 5 Laboratory of Extracellular Matrix-Rheumatology, LIM-17, Department of Medical Clinic, University of São Paulo Medical School, São Paulo 01246-903, Brazil

**Keywords:** SARS-CoV-2, neutrophils, NETs, resveratrol, supplement, antioxidant

## Abstract

The formation of microthrombi in lung autopsies indicates the involvement of NETs in the immunopathogenesis of severe COVID-19. Therefore, supplements inhibiting NET formation, in association with drugs with fewer adverse effects, should be a relevant strategy to attenuate the disease. Resveratrol (RESV) is a natural polyphenol with an important antiviral and antioxidant role. To modulate neutrophils from patients infected with SARS-CoV-2, we evaluated the in vitro effect of RESV on NET formation. Herein, we investigated 190 patients hospitalized with moderate, severe, and critical symptoms at Hospital das Clínicas, Brazil. We observed that neutrophilia in patients with severe COVID-19 infection is composed of neutrophils with activated profile able to release NET spontaneously. Notably, RESV decreased the neutrophil-activated status and the release of free DNA, inhibiting NET formation even under the specific PMA stimulus. At present, there is no evidence of the role of RESV in neutrophils from patients with COVID-19 infection. These findings suggest that adjunctive therapies with RESV may help decrease the inflammation of viral or bacterial infection, improving patient outcomes.

## 1. Introduction

The disease caused due to infection by severe respiratory syndrome coronavirus 2 (SARS-CoV-2), known as coronavirus disease 2019 or COVID-19, was declared a global pandemic by the World Health Organization (WHO) in March 2020 [1]. In June 2022, more than 500 million people had been infected worldwide, with approximately 6300 deaths. In Brazil, there were more than 31 million confirmed cases on the same date [2].

In most affected individuals, the disease presents with mild to moderate symptoms. However, a proportion of the affected population develops the most severe form of this illness, known as severe acute respiratory syndrome (SARS), requiring hospitalization. These patients suffer from pulmonary inflammation with microthrombi formation and require invasive respiratory support [3].

Some of the main characteristics observed in patients affected by SARS-CoV-2 are neutrophilia, lymphopenia, and a higher neutrophil/lymphocyte ratio linked to disease severity [4]. The impact of circulating neutrophil activation has increased interest in understanding the role of neutrophils in SARS-CoV-2 infection. The production of neutrophil extracellular traps (NETs) is spontaneous. This mechanism in the pulmonary microvessels of patients increases NET markers associated with illness severity and the ability of the plasma from patients to induce their formation in the neutrophils of healthy individuals [5].

Moreover, a disturbance in the oxidative pathway has been observed in COVID-19 patients. The increment of reactive oxygen species (ROS) intensifies the cytokine storm, coagulation, thrombus formation, and tissue hypoxia [6]. ROS generation in viral infections induces a redox imbalance for survival and replication in the host [7]. In SARS-CoV-2 infection, excessive generation of ROS harms cells, leading to the production of pro-inflammatory cytokines and ROS [6].

Therapy with antioxidant drugs may be a promising strategy to alleviate complications resulting from redox imbalance in patients affected by SARS-CoV-2 or other inflammatory viral infections. Among the various antioxidants, resveratrol draws attention for exerting other mechanisms of action, such as anti-inflammatory immunomodulatory antiviral effects.

Resveratrol (RESV) is a natural polyphenol present in red grapes, cranberries, blueberries, peanuts, soy, and red wine, with grapes having the highest content [8]. RESV is quickly metabolized in the liver, and in plasma, it binds lipoproteins and albumin, and this facilitates its entry into cells [9]. Native (non-metabolized) RESV may reach maximum peak plasma concentration 30–90 min after oral intake. The liver metabolizes RESV into glucuronide and sulfate forms. In the case of administration of a higher dose of RESV (5 g), the peak plasma concentration is estimated to be about 2.3 μM, showing low bioavailability of native resveratrol.

RESV is an antioxidant of natural origin. It has been shown to act as an antiviral and plays a role in immune stimulation, inhibiting the release of pro-inflammatory cytokine and reducing lung injury by reducing oxidative stress [10]. Antiviral effects of RESV have been observed by the in vitro inhibition of HCoV-229E and SARS-CoV-2 replication [11,12], influenza virus [13], hepatitis C [14], smallpox [15], and HIV-1 [16]. RESV activates SIRT1 signaling, possibly responsible for inhibiting viral replication. SIRT1 activates the ADAM17 enzyme (a disintegrin and metalloprotease domain 17) or converts TNF-α (TACE), suppressing the release of TNF-α and IL-6 [8].

Furthermore, both RESV and its structural analogue pterostilbene demonstrated an antiviral effect on human primary bronchial epithelial cells from healthy subjects 48 h after SARS-CoV-2 infection in vitro [17].

Pathophysiology of severe COVID-19 is marked by altered neutrophil abundance and functionality. Analyzing the effect of RESV on neutrophils from patients with COVID-19 became crucial to understanding their role in that pathogenesis. Herein, we observed an activated neutrophil population, including an immature population, in the case of acute SARS-CoV-2 infection. We showed for the first time that RESV downmodulates NET formation by decreasing the cell’s free DNA (cf-DNA). These protective in vitro effects by RESV provide options for better management of inflammation as strategies in patients with viral infection or chronic inflammatory diseases.

## 2. Materials and Methods

### 2.1. Casuistic

Peripheral blood samples were taken from patients diagnosed with COVID-19 hospitalized in Hospital das Clínicas da Faculdade de Medicina da Universidade de São Paulo. EDTA blood samples from the Central Laboratory Division (DLC) of the HC-FMUSP were kept at 4 °C and used the following day for flow cytometry analysis. We analyzed the sampled heparin tubes the day after collection, for this collect all patients authorized their participation in the study and the collection of their samples Patients were diagnosed with COVID-19 by nasopharyngeal detection of SARS-CoV-2 RNA using reverse transcriptase polymerase chain reaction (RT-PCR). The study excluded patients who were injured, those over 75 years old, or those without positive results for SARS-CoV-2.

Mild convalescent patients with COVID-19 developed non-severe symptoms and did not require oxygen therapy, oxygen by mask, or nasal cannula. Severe patients were hospitalized under non-invasive ventilation or with high-flow oxygen. Critically symptomatic patients received invasive mechanical ventilation, according to the WHO (2020). Samples were collected from May 2020 to October 2021. Healthy controls (HCs) without SARS-CoV-2 infection were also recruited.

In some experiments, we analyzed the severe and critical groups together due to the homogeneity of the data of these patients. However, before clubbing the data, we analyzed the independent groups and observed no differences.

### 2.2. Flow Cytometry for Cellular Phenotyping

Flow cytometry analyses were performed using 0.1 mL of whole blood in EDTA. Samples were incubated with antibodies CD66b V450, CD15 APC, CD16 APCY-cy7, CD14 FITC (BD Biosciences, San Diego, CA, USA), CD11b PE, and CD10 Pe-cy7 (ExBio, Vestec, Czech Republic) for 20 min at room temperature. Subsequently, they were washed and incubated with the antibodies for 60 min at 4 °C. RBCs were then lysed with FACS Lysing reagent (BD Biosciences, San Diego, CA, USA) for 15 min at room temperature. After the samples were washed, we performed LSR Fortessa flow cytometry (BD Biosciences, San Diego, CA, USA). We evaluated approximately 100,000 events per tube. We used the fluorescence minus one (FMO) staining strategy, which refers to labeling the sample with all antibodies (Ab) minus the Ab we wanted to analyze. The software applied for analysis was FlowJo™, and for T-Distributed Stochastic Neighbor Embedding (tSNE), we used an algorithm for clustering based on CD66b+ cells. The algorithm makes it possible to visualize high-dimensional flow cytometry datasets in a reduced dimensional data space. To identify and characterize clusters, we employed the self-organizing flow map (FlowSOM), setting the number of meta-clusters to 8.

### 2.3. Measurement of Oxidative Burst

To assess the oxidative burst of neutrophils, we operated dihydrorhodamine123 (DHR123, Sigma Aldrich, Darmstadt, Germany) and phorbol 12-myristate 13-acetate (PMA) (Sigma Aldrich, Darmstadt, Germany). In all, 500 µL of peripheral blood in EDTA was RBC lysed, washed, and incubated with 5 pM of DHR for 5 min at 37 °C in a 5% CO2 atmosphere. Then, we added 50 nM of PMA and left the mixture for 15 min at 37 °C. Afterward, samples were incubated with antibodies against CD15 (APC, BD Biosciences, San Diego, CA, USA) and CD10 (APC-Cy7, ExBio) for 20 min at room temperature. Cells were washed and fixed in solution, and LSR Fortessa flow cytometry (BD Biosciences, San Diego, CA, USA) was performed. Approximately 500,000 events were obtained and analyzed with the FlowJo™ software (v.10.8.1).

### 2.4. Neutrophil Enrichment and Culture with Resveratrol

Peripheral blood collected in sodium heparin was half diluted in 6% Dextran at 37 °C in a 5% CO_2_ atmosphere. The supernatant was collected and centrifuged on Ficoll–Hypaque solution (1077 density). Afterward, we discarded the plasma and the cells were RBC lysed with ACK (Gibco, New York, NY, USA) for 5 min and centrifuged. We diluted cells in RPMI medium without phenol red (Gibco, New York, NY, USA), and the obtained neutrophils, enriched by 88%, were checked by flow cytometry. Neutrophils were diluted in RPMI medium without phenol red at 200,000 cells/well of 24-well plates (Jet Biofil, Guangzhou, China) under 13 mm round coverslips, incubated with 100 µM resveratrol (Sigma-Aldrich, Darmstadt, Germany), and dissolved in acetone 50 mg/mL. The cells were kept for 30 min at 37 °C under a 5% CO_2_ atmosphere. We stimulated the cells with 50 nM of PMA for 4 h at 37 °C. After incubation, the supernatant was kept for protein analysis and NET quantification and the neutrophils on the coverslip were fixed with 4% formaldehyde for the fluorescence assay analysis.

The concentration of resveratrol was defined according to a dose–response test and assay for assessing LDH cytotoxicity. Initial experiments showed the purity of neutrophil isolation to be greater than 85%. However, due to the amount of the sample, the purity test was not performed in all assays. After the dilution (100 µM) of cells in RPMI 1640 medium without phenol red, as described above, resveratrol was solubilized in acetone according to the manufacturer’s instructions where the concentration was 50 mg/mL.

### 2.5. NET Formation by Immunofluorescence

Neutrophils on glass coverslips were stained with antibodies of mouse anti-MPO (2C7; Abcam; 1:500), rabbit anti-histone H3 (H3Cit; Abcam;1:250), rabbit anti-elastase (NE; Abcam; 1:1000), and mouse anti-PAD4 (OTI4H5; Abcam; 1:200). We washed the coverslips and incubated the neutrophils with the secondary antibodies, i.e., goat anti-rabbit IgG Alexa 568 (Abcam, 1:200) and goat anti-mouse IgG Alexa 488 (Abcam, 1:500). The nuclei were stained with Hoechst 33,342 (Abcam; 1:500) for 5 min. We mounted the coverslips on slides for analysis under a photographic camera (Olympus Co., St. Laurent, Quebec, Canada) attached to the Olympus BX-51 microscope (Olympus BX51, Olympus Co., Tokyo, Japan). The camera captured the images for digitalization (Oculus TCX, Coreco, Inc., St. Laurent, QC, Canada) and were subsequently processed by the software Image-Pro Plus 6.0. We identified NETs as the area of MPO and H3Cit localization, neutrophil activation NE, and PAD4 expression.

### 2.6. Measurements of Cytokines

Cytokine concentration in supernatants from cultures and plasma from patients was assessed by ELISA assays according to the manufacturer’s specifications. R&D Systems was used for IL-6 (DY406), IL-8 (DY208), IL-10 (DY217B), and MMP-9 (DY211), and Biolegend was used for IFN-α (446404) and IFN-γ (430104).

### 2.7. NETs Quantification by Quant-iT PicoGreen

To quantify the levels of cell-free DNA in supernatants and plasma, we applied the Quant-iT PicoGreen kit technique (Invitrogen; cat. P11496) as described by Colón et al. (2019) and Czaikoski et al. (2016).

### 2.8. Statistical Analysis

The results are expressed as the median and the interquartile range (IQR). One-way ANOVA test was used for analysis, and the non-parametric Kruskall–Wallis test was used to compare the three groups of data. We operated the Mann–Whitney test for comparative analysis of two groups and the Wilcoxon test for analysis of two paired samples. For correlation analysis, we worked with Pearson’s test. A *p*-value less than or equal to 0.05 was considered significant.

## 3. Results

### 3.1. Characteristics of the COVID-19 Patients and Healthy Controls

COVID-19 patients from Hospital das Clínicas da Faculdade de Medicina da Universidade de São Paulo (HC-FMUSP), Brazil, included 190 individuals. This group was composed of patients with mild, severe, and critical symptoms according to the classification of the WHO. Moreover, 46 non-infected individuals were added, confirmed by serology for IgG anti-SARS-CoV-2. Death was recorded in the case of 5.6% of the patients with mild, 9.5% of those with severe, and 45.9% of those with critical symptoms. Table 1 displays the demographic data. The samples used varied according to the employed assay.

**Figure 1:**
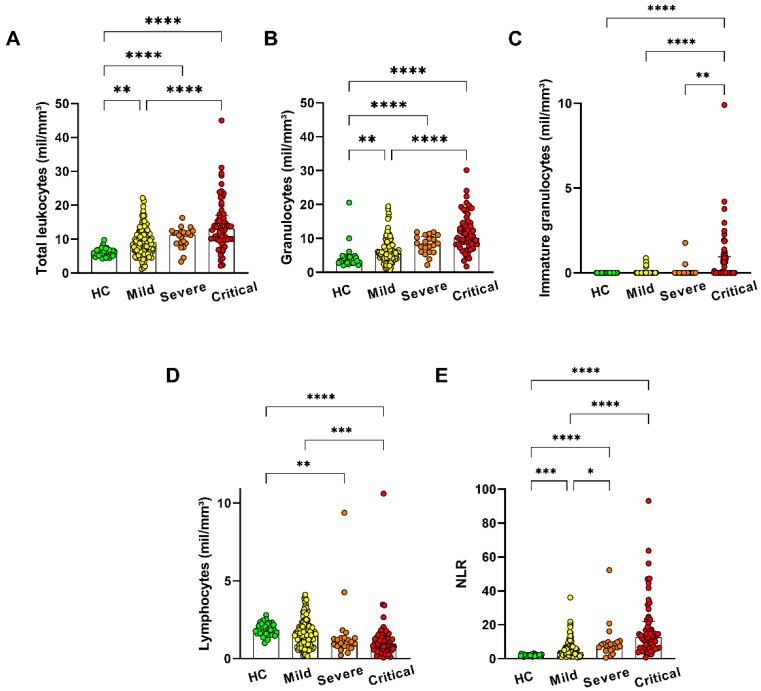
Blood neutrophilia with presence of immature neutrophils with COVID-19 infection severity. Blood count in (**A**) total leukocyte, (**B**) granulocytes, (**C**) immature granulocytes, (**D**) lymphocytes, and (**E**) the neutrophil/lymphocyte ratio (NLR) of patients with mild (*n* = 110), severe (*n* = 22), and critical (*n* = 60) symptoms and healthy controls (HC *n* = 29). The bars represent the median and interquartile range. * *p* ≤ 0.05, ** *p* ≤ 0.01, *** *p* ≤ 0.001 and **** *p* ≤ 0.0001.

In modulating the in vitro activity, the aim was to verify the phenotypic and functional aspects of neutrophils in COVID-19 patients. We analyzed the maturation degree of neutrophils in COVID-19 patients. Then, we examined activation, maturation, and degranulation expression in these molecules. Leukocytosis was observed conforming to the severity of the infection in patients (Figure 1A), due to the increment of granulocytes (Figure 1B), and with immature granulocytes (Figure 1C). As expected, the neutrophil-to-lymphocyte ratio (NLR) increased in agreement with the severity of the COVID-19 infection (Figure 1E).

**Figure 2:**
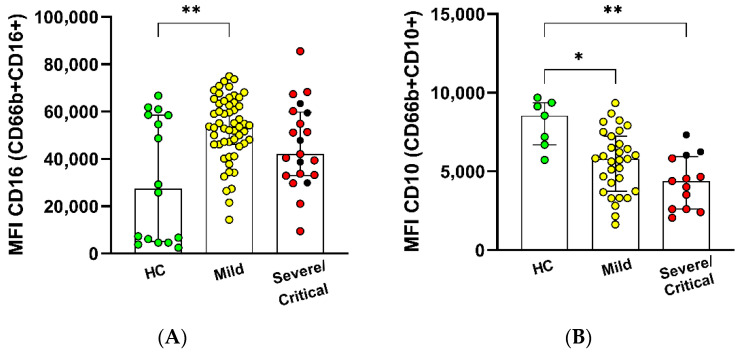
Immature neutrophil profile with COVID-19 infection severity. CD66b+ expression from patients with mild (*n* = 55), severe (*n* = 5), and critical (*n* = 16) symptoms and healthy controls (HC *n* = 16) In (**A**) CD16 and (**B**) CD10, assessed by flow cytometry are shown as MFI (median of fluorescence intensity). The bars represent the median and interquartile range. * *p* ≤ 0.05 and ** *p* ≤ 0.01.

The marker CD10 means mature neutrophils in the latest stage of differentiation, while CD33 represents immature neutrophils [18,19]. Despite no change in the percentage values, mildly infected individuals showed an elevated MFI expression of CD16+ in CD66+ (Figure S2). However, the expression of the marker CD10 in neutrophils CD66b+CD10+ decreased with the severity of the illness (Figure 2).

A similar expression was observed in CD15, CD33, CD14, and CD11b within the analyzed groups (Figure S2). The data showed that neutrophils from COVID-19 patients have a disturbing frequency, including immature neutrophils, in addition to an elevated number of mature neutrophils.

### 3.2. Cluster Analysis in Granulocyte Populations

**Figure 3:**
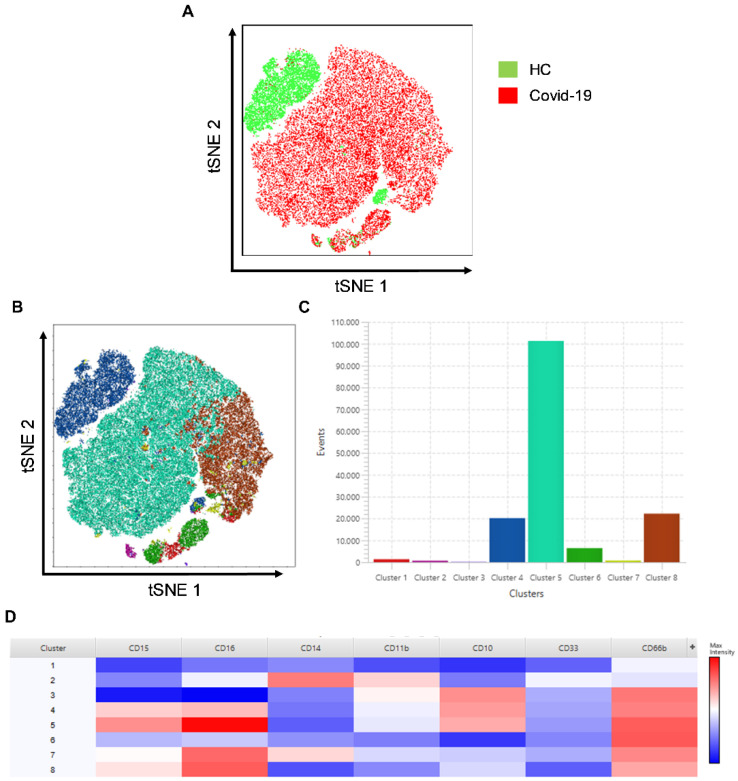
Distinct neutrophil clusters in COVID-19 patients in t-SNE. (**A**) Representation of samples from COVID-19 patients and healthy control by unified in t-SNE analysis, demonstrating difference in clustering of CD66b+ live-cell populations. (**B**) t-SNE graph with 8-cluster FlowSOM analysis. (**C**) Graph demonstrating the number of events of each cluster. (**D**) Heatmap analysis of the clusters demonstrating the cell markers.

Concerning evaluating the neutrophil clusters, we verified the t-SNE and FlowSOM, selected by gate-forward compared with side-scatter in flow cytometry. Figure 3A shows a different profile of granulocyte populations within patients with severe COVID-19 and healthy controls. Notably, when the cell populations are made to overlap, we observed that cluster 4 disappears in infected individuals. The cluster exhibits the expression of CD66+CD10+CD16lowCD15+, whose cells present characteristics of non-activated cells owing to the low expression of CD16 (Figure 3A–C).

Figure 3D shows the heatmap of the clusters located in the granulocyte populations. We can verify the high variability of subsets in the clusters. Additionally, relevant was the high frequency of clusters 7 and 8 in patients and their absence in control subjects. The heatmap allows us to observe the CD66b+CD10lowCD16highCD15+ population, which has low-density granulocytes (LDGs). The same population represents neutrophils with pro-inflammatory, suppressive, and augmented capacity for NET formation [20].

The findings of t-SNE analysis evidence that the neutrophil cluster disappears with COVID-19 infection, similar to other neutrophil populations that correspond to the severity of the disease (Figure S3).

### 3.3. High Circulants Levels of Cytokines and Increased Neutrophil Oxidative Burst in COVID-19 Patients

**Figure 4:**
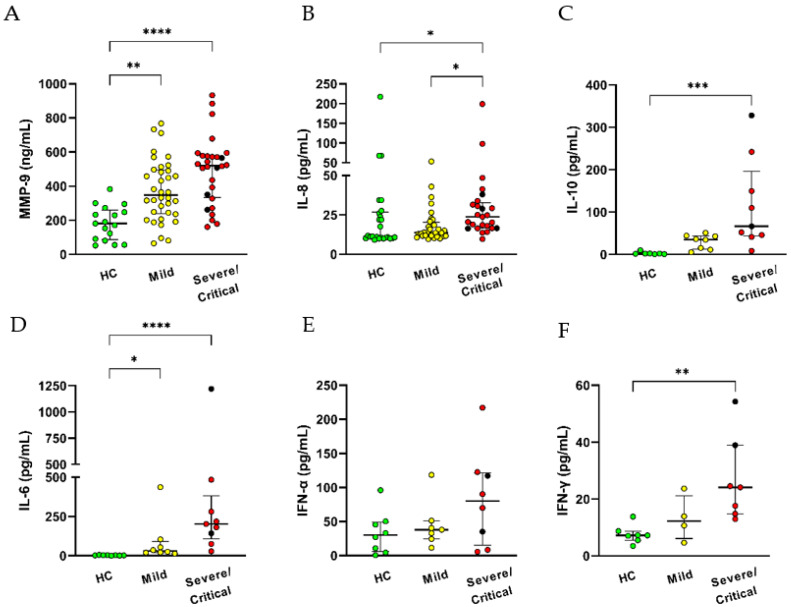
Increased pro-inflammatory cytokines levels with COVID-19 severity. Evaluation of cytokines in serum were analyzed by ELISA: (**A**) MMP-9 and (**B**) IL-8 were evaluated in healthy control HC = 17–25 and patients with Mild = 32–36, Severe = 2–4, Critical = 21–24 and (**C**) IL-10, (**D**) IL-6, (**E**) IFN-αd and (**F**) IFN-γ were evaluated in HC = 7–8, Mild = 7–8, Severe = 2, Critical = 7. Black circles are represented by severe, and red circles by critical patients. Data shows median and interquartile range. * *p* ≤ 0.05, ** *p* ≤ 0.01, *** *p* ≤ 0.001 and **** *p* ≤ 0.0001.

To begin with, we assessed IL-8, IL-10, IL-6, IFN type I (IFN-α) and type II (IFN-γ), and metalloproteinase-9 (MMP-9) in the serum of infected patients and healthy control (Figure 4A,D). Compared with the HC group, increased serum levels of MMP-9 and IL-6 were found in COVID-19 patients regardless of the disease severity. Other cytokines, such as IL-8, IL-10, and IFN-γ, were detected at augmented levels only in severe/critical patients (Figure 4B,C,F). These and other pro-inflammatory factors may contribute to the activated status of neutrophils and other innate cells in COVID-19 infection.

Furthermore, we assessed the oxidative burst using the measurement of oxidation of the DHR 123 assay to analyze the neutrophil function. Data represent the oxidative burst index (OBI) of neutrophils, the ratio of the MFI (the MFI of stimulated cells minus the MFI of unstimulated cells), and oxidative percentage (value of the DHR of stimulated minus unstimulated cells).

**Figure 5:**
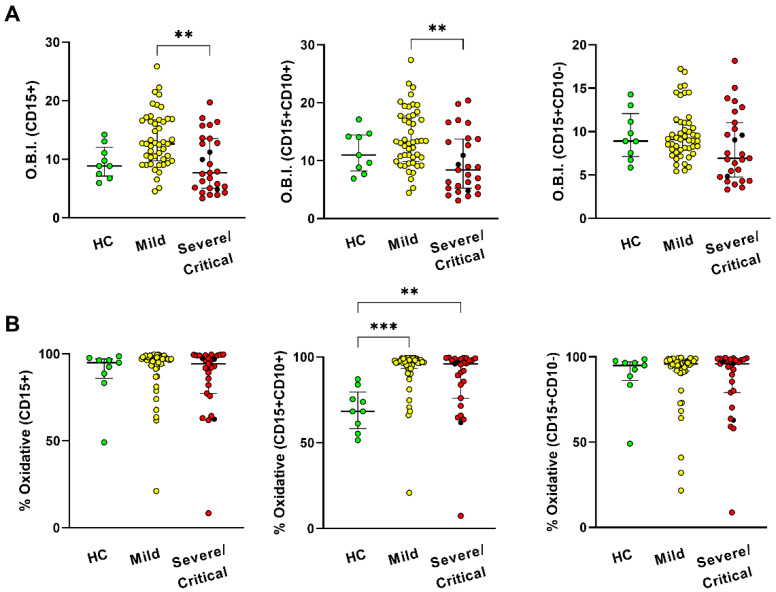
CD15+CD10+ neutrophils with increased respiratory burst in mild COVID-19 infection. DHR oxidation assay was analyzed in neutrophils from patients with COVID-19 (moderate (*n* = 47), severe (*n* = 3), and critical (*n* = 24)), compared with healthy subjects (HC, *n* = 9) by flow cytometry. (**A**) Index of the data of Oxidative Burst Index (O.B.I) (**B**) oxidative percentage. CD15+CD10+ cells represent mature neutrophils and CD15+CD10- cells that represent immature neutrophils. Data show the median and interquartile range. ** *p* ≤ 0.01 and *** *p* ≤ 0.001.

Neutrophils in the group with mild symptoms had a large OBI by CD15+ population and CD15+CD10+ levels compared to the groups with severe/critical symptoms (Figure 5A). Apart from this, neutrophils increased the oxidative percentage of CD15+CD10+ in both mildly ill and severely/critically ill patients in contrast to the HC group. Neither the OBI nor the percentage presented oxidative changes in CD15+CD10- cells within the study groups.

### 3.4. Resveratrol Downmodulates NET Formation in Neutrophils from COVID-19 Patients

For in vitro assays, we used 100 μM of RESV, that is, a 40-fold increase in the native RESV after high RESV oral intake. This concentration was previously established in laboratorial assays based on the RESV (0–100 μM) ability to inhibit in vitro LPS-induced inflammatory markers in monocytes [21] as well as to inhibit the SFKs–Btk–Vav pathway in human neutrophils (10–50 μM) [22].

RESV has broad antioxidant and other bioactive activities. The bioactive activities include anti-inflammatory, anticarcinogenic, cardioprotective, phytoestrogen, and neuroprotective functions [23]. We evaluated the action of RESV on the NET formation by neutrophils from COVID-19 patients.

**Figure 6:**
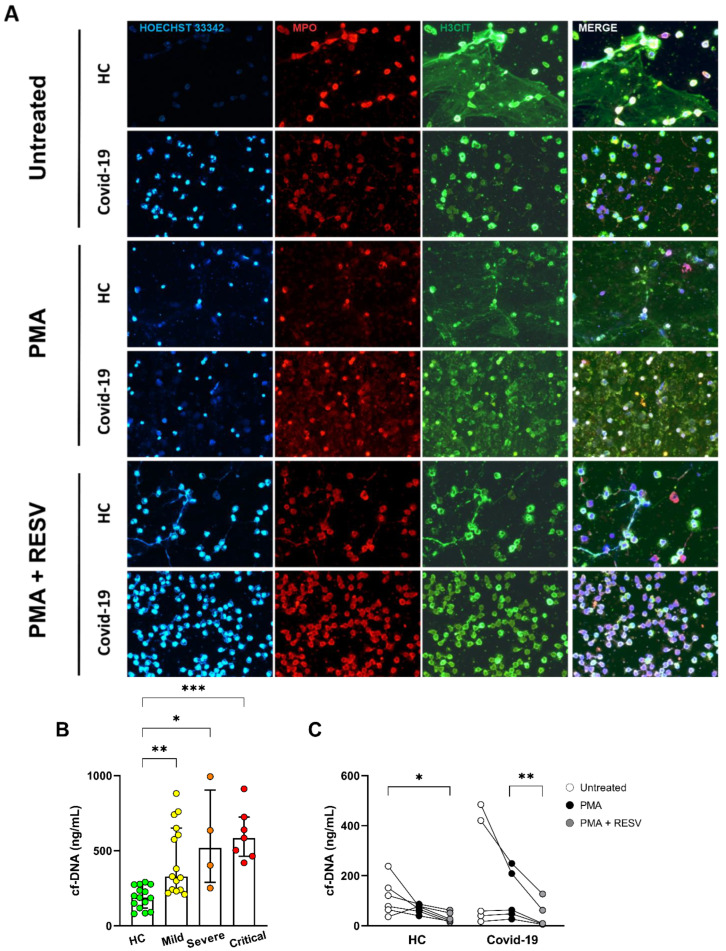
Resveratrol is able to modulate NET formation. (**A**) Representative immunofluorescence analysis of NETs release by neutrophils isolated from healthy controls and COVID-19 patient, cultured for 4 h at 37 °C. Cells were stained for nuclei (Hoechst 33342, blue), MPO (red), and H3Cit (green). Original magnification 40x. (**B**) Determination of cell-free DNA (cf-DNA) in plasma from control subjects (*n* = 15) and SARS-CoV-2 infected patients (Mild = 13; Severe = 4, Critical = 9) by Quant-iT PicoGreen. (**C**) Cell-free DNA in the supernatant of enriched neutrophil culture incubated with 100 µM resveratrol 30 min and stimulated with PMA 4 h of healthy (*n* = 6) and SARS-CoV-2 infected (*n* = 5) subjects. Data represent the median and interquartile range. * *p* ≤ 0.05, ** *p* ≤ 0.01 and *** *p* ≤ 0.001.

Figure 6A shows a panel of immunofluorescence analysis of NETs released by neutrophils isolated from the peripheral blood of healthy controls and COVID-19 patients. At the basal level, we observed a pronounced presence of MPO and H3Cit that enriched co-localization by neutrophils from COVID-19 patients. PMA stimulation seems to decrease the number of cells and expression of markers, but treatment with RESV leads to an increase in cell number and viability.

Next, we analyzed an increased amount of cell-free DNA in plasma from patients with COVID-19 compared to HC (Figure 6B). To verify the in vitro RESV effect, we assessed the DNA liberation of neutrophils upon PMA stimulation. Figure 6C shows that RESV reduced the amounts of DNA in the supernatants of neutrophils from COVID-19 patients, demonstrating that the antioxidant effect of RESV minimizes NET formation.

**Figure 7:**
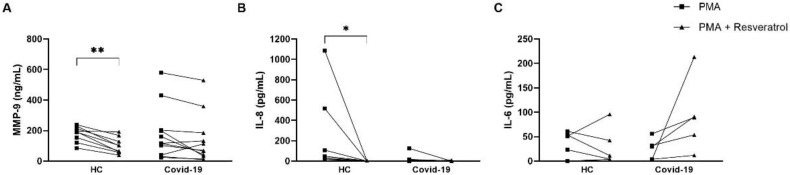
Resveratrol downmodulates cytokine production in PMA-activated neutrophils of healthy donors. Neutrophils (2 × 105 cells/mL) were stimulated with PMA, in presence or not with resveratrol 100 µM, from healthy controls (*n* = 6–9) and severe COVID-19 patients (*n* = 5–11). Supernatants of the cultures were determined for (**A**) MMP-9, (**B**) IL-8, and (**C**) IL-6 by ELISA. * *p* ≤ 0.05, ** *p* ≤ 0.01.

We analyzed the in vitro RESV effect cytokines production. The effect of RESV was observed in healthy subjects but not in patients where there was a decrease in MMP-9 and IL-8 but not in IL-6 (Figure 7).

## 4. Discussion

In our study, we verified a novel role of the flavonoid RESV in COVID-19 infection, shown to decrease the release of free DNA from neutrophils, even when in vitro stimulation with PMA reduces NET generation. This finding demonstrates that RESV attenuates NET formation and the activated profile. Concomitantly, it increases the viability of neutrophils in patients with severe COVID-19 infection, observed through immunofluorescence. The augmentation was revealed by diminished MPO and citrullinated histone H3, a marker of extracellular trap from neutrophils.

The potent RESV antioxidant action was revealed to prevent oxidative damage in neutrophils, improving their viability. Despite the modulatory effects of RESV on NET generation, it can downregulate MMP-9 and IL-8 secretion after PMA stimulation in samples from healthy subjects. However, the same effect was not observed in individuals with COVID-19. This evidences that the neutrophils from COVID-19 patients are already activated, mainly under severe conditions. However, the pre-formed components, such as MMP-9, were not modulated by RESV. Furthermore, it can corroborate the baseline condition of neutrophils from infected individuals, which spontaneously release NET. Neutrophils have several pre-formed effector-protein-like cytokines, such as IL-6 and IL-8, and allow immediate antimicrobial functions without needing the time for a new synthesis cycle [24].

Up to this point, there have only been theoretical discussions about using RESV in COVID-19 due to its ability to cleave DNA in NET formation, thus improving lung function during acute airway infections [25]. In addition, it is speculated that RESV used as an NRF2 activator may act as an antioxidant while inhibiting the oxidative stress response and NET formation [26]. Indeed, our findings endorse the in vitro action of RESV in inhibiting NET formation, declining the release of free DNA from neutrophils of patients with severe COVID-19. It seems that RESV inhibits the NETosis process rather than the cleavage of DNA during NET formation.

In the pathophysiology of COVID-19, the neutrophilia and a pronounced immaturity and increase in ROS are notable and result in increased heterogeneity in the pool of circulating neutrophils. The enlargement of CD10 negative neutrophils marks immaturity, and we also observed that an increase in immature neutrophils is correlated with disease severity. For critically ill patients, there is a sign of bone marrow stimulation through the presence of left shift and myeloid lineage precursors, similar to diminished CD10 expression. Phenotypically, low-density granulocyte (LDG) cells differentiate through the expression of CD16. Intermediate or low CD16 presence is usual in immature cells, while high CD16 expression characterizes mature LDGs.

Despite the presence of immature cells in the peripheral blood of patients with severe COVID-19, the T-SNE analyses represent the presence of CD16high and CD16interm neutrophils, which correspond to mature LDGs. It has been described that critically ill patients possess CD16interm LDGs and that these cells have a greater capacity for spontaneous NET formation and phenotypic characteristics of activated neutrophils [20]. We observed that in patients predominantly cluster 5 is represented by CD66b+CD10+CD16highCD15+ expression, which may be associated with activated cells in acute COVID-19 infection.

Moreover, in patients, we noticed mature LDGs in two clusters, clusters 7 and 8. In contrast, in healthy controls, mature LDGs were distinguished predominantly in cluster 4, with low expression of CD16 (CD66+CD10+CD16lowCD15+). Although CD16 expression is linked to the maturation factor, the data show that the lower expression of CD16 may also be related to the activation status.

We observed that the LDG CD16interm cell profile is associated with the pro-inflammatory profile in patients with severe COVID-19 and increased ex vivo activated neutrophils with a high capacity for spontaneous NET formation. The data indicate the release of genetic material into the extracellular environment due to NETosis. The pro-NETotic capability of serum from individuals hospitalized with COVID-19 is already well established [27].

The dosage of cf-DNA was performed from the neutrophil culture supernatant. For storage of the supernatant, we used the culture medium in which the neutrophils were incubated together with the coverslips that would later be sent for immunofluorescence; however, we observed in our immunofluorescence assay that in the PMA condition there is staining with proteins and DNA at the bottom of the slide, and we noticed a modulation by resveratrol that resembles the baseline condition. The discrepancy with the lower PMA in relation to the basal one may have occurred due to the high adherence of cells and proteins in the coverslip and lower capture in the supernatant.

The high plasma levels seem to result in spontaneous NETosis derived from LDGs and lead to complications such as thrombosis and tissue damage in patients with a worse prognosis. In addition to the neutrophils, the sources of cf-DNA in COVID-19 can be derived from vascular endothelium, hepatocytes, adipocytes, kidney, heart, and lung and may cause tissue injury [28].

The dosage of cf-DNA was performed from the neutrophil culture supernatant. For storage of the supernatant, we used the culture medium in which the neutrophils were incubated together with the coverslips that would later be sent for immunofluorescence. We observed in our immunofluorescence assay that in the PMA condition there is staining with proteins and DNA at the bottom of the coverslips, and we noticed a modulation by RESV that resembles the baseline condition. The discrepancy with the lower PMA in relation to the basal one may have occurred due to the high adherence of cells and proteins in the coverslip and lower capture in the supernatant.

In addition, high circulating levels of, for example, MMP-9, IL-6, and IL-8 are detected mainly in COVID-19 critical patients. High expression of neutrophil-related cytokines IL-8 and IL-6 in serum and neutrophilia has been described as a predictor of poor outcome, contributing to a cytokine storm and the further development of ARDS and organ failure in COVID-19 patients. The secretion of MMP-9 is associated with the risk of pulmonary inflammatory disease and a worse prognosis in ICU individuals [29]. Furthermore, degranulated prothrombotic neutrophil phenotypes in peripheral blood demonstrate upregulation of pathways. These pathways are connected with the response to and production of IL-8 [30]. The results suggest that the elevation of MMP9 linked with IL-8 may contribute to neutrophil migration to the lung. An experimental model of influenza evidenced MMP9 pathogenesis due to excessive neutrophil migration into the respiratory tract in response to viral replication [31].

Peculiarly, the anti-inflammatory IL-10 increased only in severe/critical patients. The marker with IL-6 is established as a predictor of non-severe or severe state in SARS-CoV-2 infection [32]. Neutrophils are significant contributors of IL-10 at the site of infection during sepsis [33]. However, whether neutrophils can also contribute to augmenting circulating IL-10 levels in COVID-19 has to be explored.

## 5. Conclusions

Our study generated novel data on RESV effects on neutrophils from COVID-19 patients. The data demonstrate promising results for antioxidant effects and the inhibition of NET formation. An approach aimed at inhibiting NET formation or attenuating neutrophil activation may be beneficial and find wide use in bacterial infections and/or inflammatory diseases.

## Figures and Tables

**Table 1:** Demographic table.

	Uninfected		Mild		Severe		Critical	
	*n* = 46		*n* = 108		*n* = 21		*n* = 61	
	Mean	SD	Mean	SD	Mean	SD	Mean	SD
Gender Male/Female	16/30		65/43		14/7		37/24	
Age-Male	47	12.1	54	13.2	51	12.5	56	10.6
Age-Female	39	11.5	54	14.7	56	11.8	54	16.2
Evolution			*n*	%	*n*	%	*n*	%
Discharge			92	85.2	18	85.7	29	47.5
Death			6	5.6	2	9.5	28	45.9
Transfer to another institute			10	9.3	1	4.8	4	6.6
Comorbidities			*n*	%	*n*	%	*n*	%
HAS			56	51.9	11	52.4	33	54.1
DM			45	41.7	8	38.1	19	31.1
Neoplasms			22	20.4	2	9.5	4	6.6
Vascular diseases			10	9.3	1	4.8	7	11.5
Obesity			24	22.2	6	28.6	18	29.5
Use of alcohol and cigarettes			32	29.6	5	23.8	11	18.0
Transplants			10	9.3	0	0.0	4	6.6
Kidney diseases			15	13.9	0	0.0	4	6.6
Gastrointestinal/Liver diseases			7	6.5	1	4.8	4	6.6
Heart diseases			28	25.9	4	19.0	8	13.1
Neurological diseases			9	8.3	0	0.0	4	6.6
Respiratory diseases			15	13.9	0	0.0	6	9.8
Metabolic/autoimmune diseases			34	31.5	5	23.8	15	24.6
Other infectious diseases			9	8.3	0	0.0	2	3.3
Other diseases			10	9.3	3	14.3	9	14.8
No comorbidities			5	4.6	4	19.0	7	11.5
1			16	14.8	4	19.0	11	18.0
2			21	19.4	4	19.0	20	32.8
3			25	23.1	4	19.0	9	14.8
≥4			41	38.0	5	23.8	14	23.0
Hospitalization history			Mean	SD	Mean	SD	Mean	SD
Days of symptoms until collection			23.4	20.7	14.7	5.0	18.7	9.4
Positive COVID-19 test days until collection			15.8	19.7	7.5	4.4	10.2	8.8
Hospitalization days until collection			16.3	20.0	5.3	2.8	9.5	8.8
Days from collection to completion			11.3	16.9	15.0	10.0	27.4	25.4
Total days of hospitalization			27.6	27.8	20.3	10.8	36.9	26.5

Table 1 Demographic data of the COVID-19 patient cohort and control subjects used in the study. Data on age, evolution, and comorbidities of control subjects and COVID-19 patients classified as moderate, severe, and critical. M–male; F–female; SC–healthy control; n–sample number; SAH–systemic arterial hypertension; DM–diabetes mellitus.

## Data Availability

Not applicable.

## Supplementary Materials

The following supporting information can be downloaded at: https://www.mdpi.com/article/10.3390/antiox11091690/s1, Figure S1: Strategy of the immunophenotyping of neutrophils in peripheral blood; Figure S2: Expression of CD15, CD11b, CD14, and CD33 on the CD66+ cells; Figure S3: Analysis of t-SNE shows marker expression and distribution of neutrophils populations in healthy subjects (HC) and in patients with COVID-19 according disease severity; Figure S4: Resveratrol is able to modulate activation stage by neutrophils.
