# Supplementary material for: Resveratrol Downmodulates Neutrophil Extracellular Trap (NET) Generation by Neutrophils in Patients with Severe COVID-19
Source: Antioxidants (Basel). 2022 Aug 29;11(9):1690. doi: 10.3390/antiox11091690 (PMC9495554; doi:10.3390/antiox11091690)
Supplement: Supplementary file 1 [file antioxidants-11-01690-s001.zip › antioxidants-1855611-supplementary.pdf]

antioxidants

Supplementary Materials

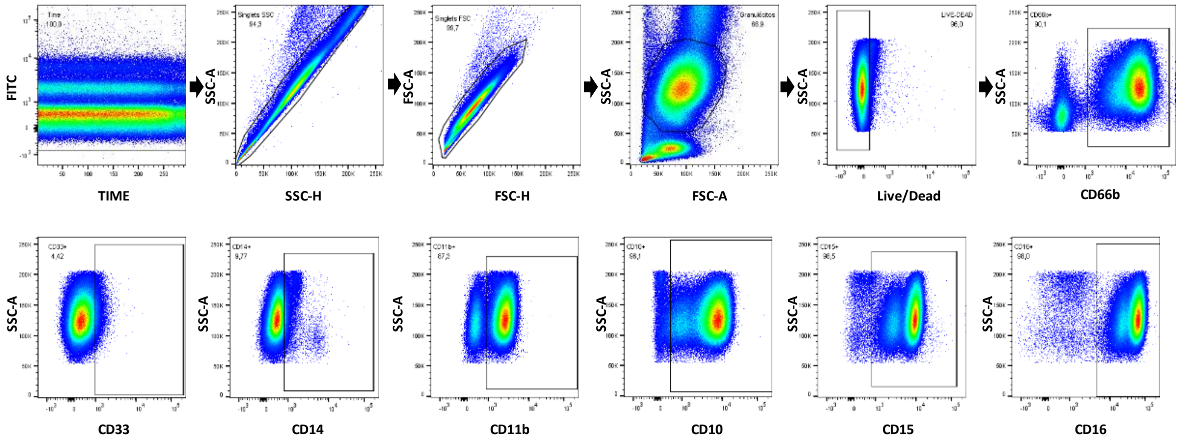

Figure S1. Strategy of the immunophenotyping of neutrophils in peripheral blood. For this the Time was evaluated, to remove interferences from possible laser oscillations, then the singlets were selected for SSC and FSC, then by FSC and SSC we selected the granulocyte population. After selection of live cells, CD66b population, which includes granulocytic cells was selected. From this gate, we evaluated the expression of markers CD33, CD14, CD11b, CD10, CD15 and CD16 in healthy individuals, patients with mild, severe and critical symptoms.

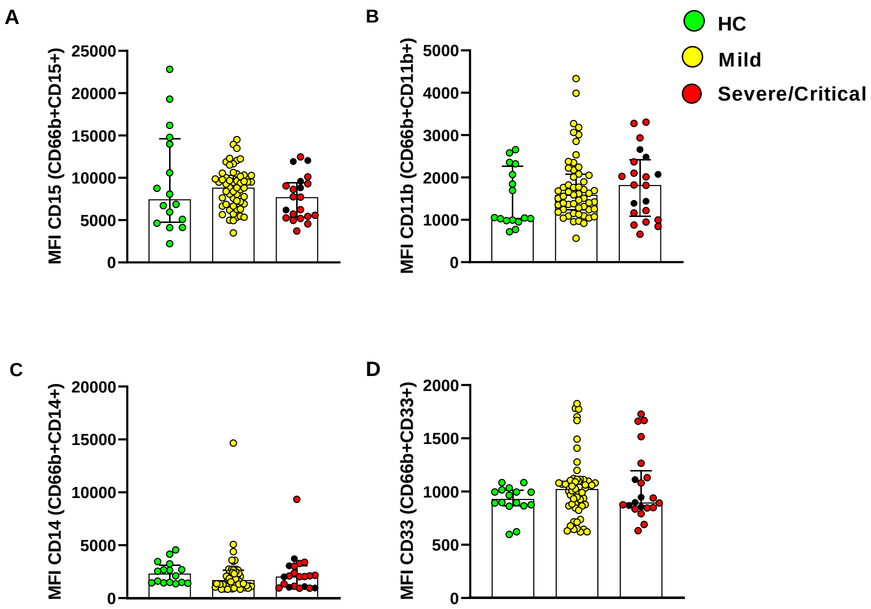

Figure S2. Expression of CD15, CD11b, CD14 and CD33 on the CD66+ cells. Data shows MFI (median of fluorescence intensity) of markers in CD66b+ by flow cytometry of patients with mild (n = 55), severe (n = 5) and critical (n = 16) symptoms and healthy controls (HC n = 16) In (**A**) CD15, (**B**) CD11b, (**C**) CD14 and (**D**) CD33. The bars represent the median and interquartile range. $^{*}p \leq 0.05$, $^{**}p \leq 0.01$, $^{***}p \leq 0.001$ and $^{****}p \leq 0.0001$.

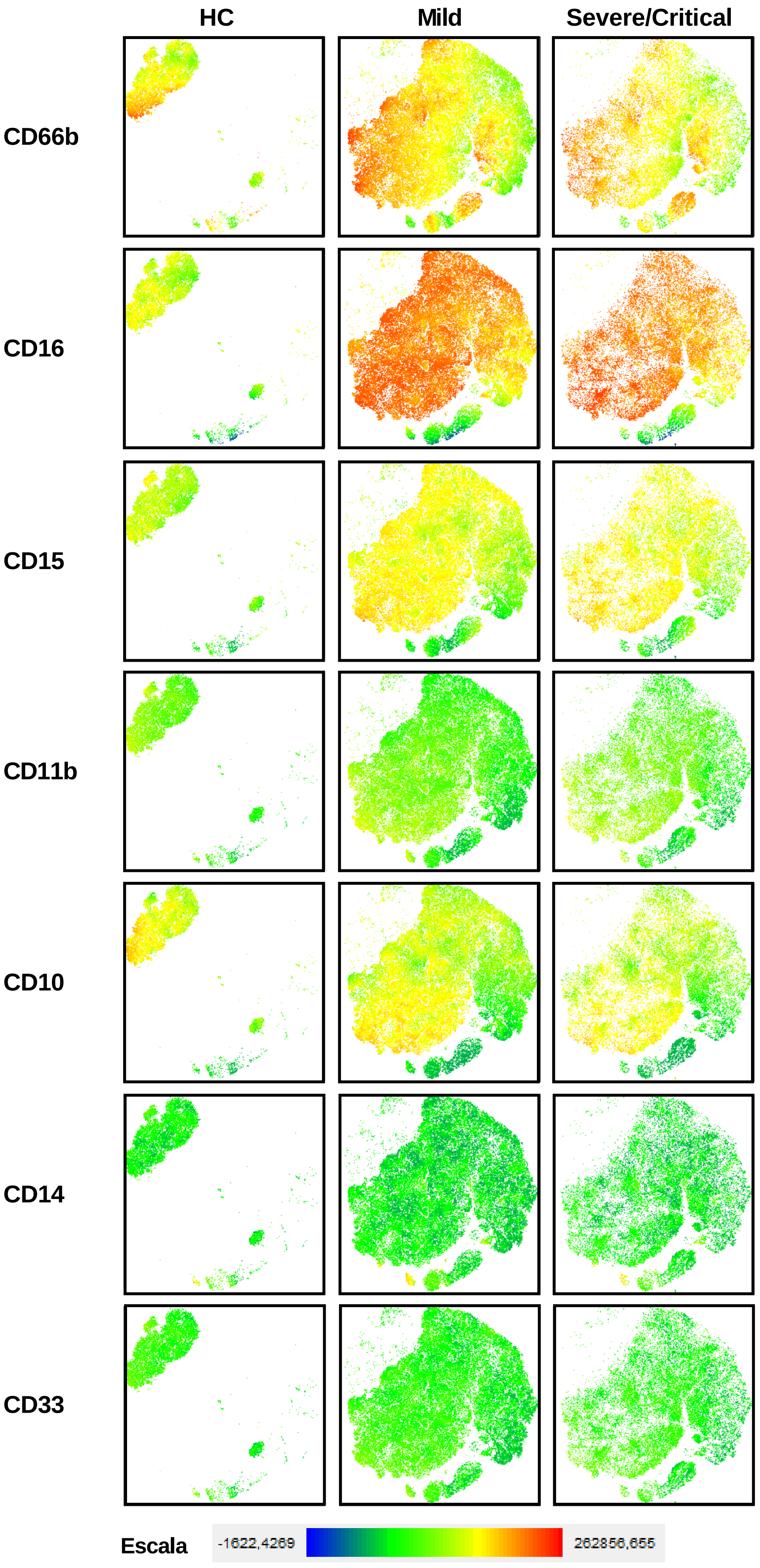
HC
Mild
Severe/Critical
CD66b
CD16
CD15
CD11b
CD10
CD14
CD33
Escala
-1622,4269
262856,655

Figure S3. Analysis of t-SNE shows marker expression and distribution of neutrophils populations in healthy subjects (HC) and in patients with COVID-19 according disease severity.

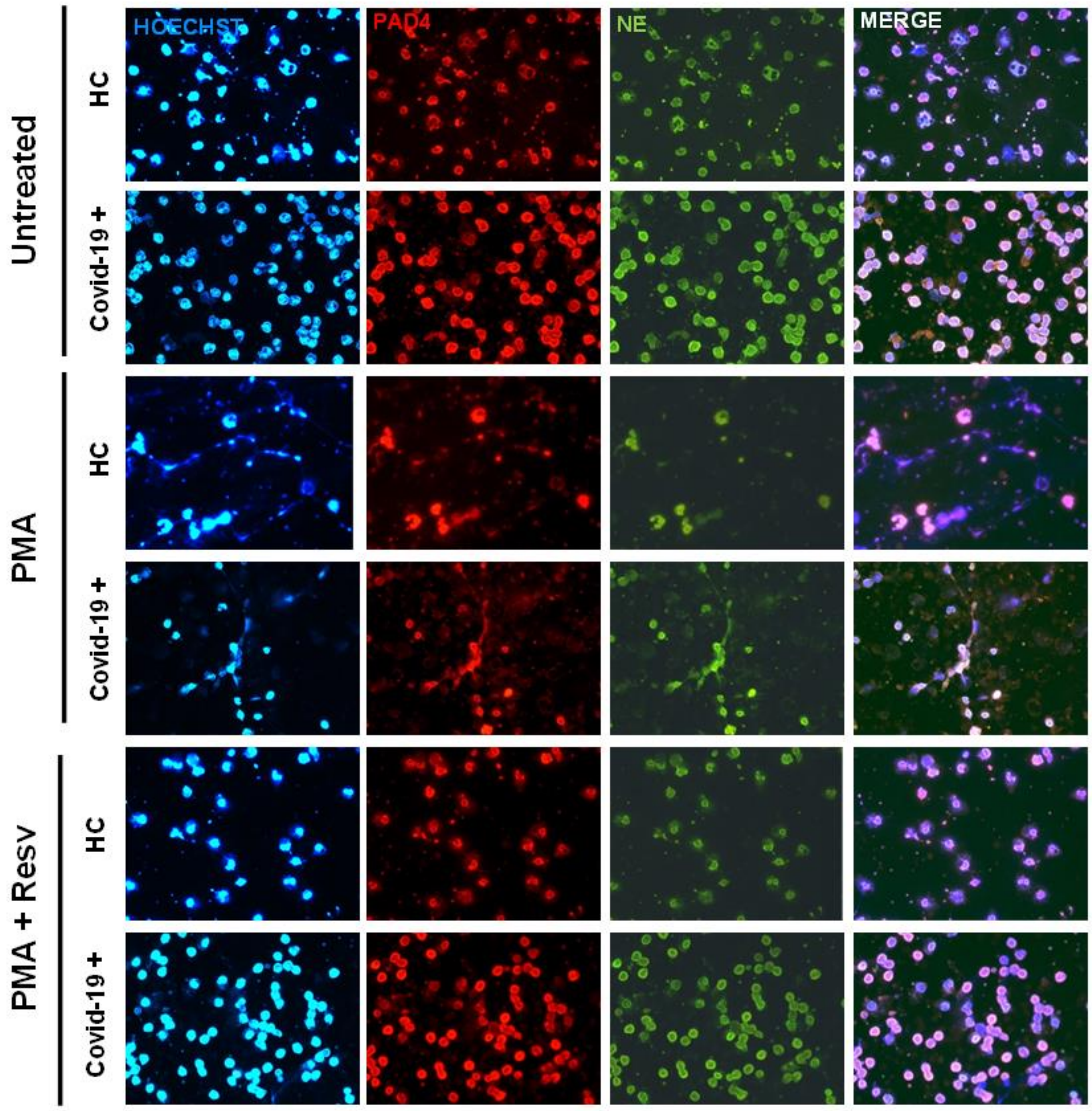

Figure S4. Resveratrol is able to modulate activation stage by neutrophils. Representative immunofluorescence analysis activation stage by neutrophils isolated from healthy controls and COVID-19 patient, cultured for 4 h at 37°C. Cells were stained for nuclei (Hoechst 33342, blue), PAD4 (red), and NE (green). Original magnification 40x.
